# Combining Nonclinical Determinants of Health and Clinical Data for Research and Evaluation: Rapid Review

**DOI:** 10.2196/12846

**Published:** 2019-10-07

**Authors:** Elizabeth Golembiewski, Katie S Allen, Amber M Blackmon, Rachel J Hinrichs, Joshua R Vest

**Affiliations:** 1 IUPUI Richard M Fairbanks School of Public Health Indianapolis, IN United States; 2 Regenstrief Institute, Inc Indianapolis, IN United States; 3 IUPUI University Library Indianapolis, IN United States

**Keywords:** social determinants of health, socioeconomic factors, inequalities, population characteristics, social conditions

## Abstract

**Background:**

Nonclinical determinants of health are of increasing importance to health care delivery and health policy. Concurrent with growing interest in better addressing patients’ nonmedical issues is the exponential growth in availability of data sources that provide insight into these nonclinical determinants of health.

**Objective:**

This review aimed to characterize the state of the existing literature on the use of nonclinical health indicators in conjunction with clinical data sources.

**Methods:**

We conducted a rapid review of articles and relevant agency publications published in English. Eligible studies described the effect of, the methods for, or the need for combining nonclinical data with clinical data and were published in the United States between January 2010 and April 2018. Additional reports were obtained by manual searching. Records were screened for inclusion in 2 rounds by 4 trained reviewers with interrater reliability checks. From each article, we abstracted the measures, data sources, and level of measurement (individual or aggregate) for each nonclinical determinant of health reported.

**Results:**

A total of 178 articles were included in the review. The articles collectively reported on 744 different nonclinical determinants of health measures. Measures related to socioeconomic status and material conditions were most prevalent (included in 90% of articles), followed by the closely related domain of social circumstances (included in 25% of articles), reflecting the widespread availability and use of standard demographic measures such as household income, marital status, education, race, and ethnicity in public health surveillance. Measures related to health-related behaviors (eg, smoking, diet, tobacco, and substance abuse), the built environment (eg, transportation, sidewalks, and buildings), natural environment (eg, air quality and pollution), and health services and conditions (eg, provider of care supply, utilization, and disease prevalence) were less common, whereas measures related to public policies were rare. When combining nonclinical and clinical data, a majority of studies associated aggregate, area-level nonclinical measures with individual-level clinical data by matching geographical location.

**Conclusions:**

A variety of nonclinical determinants of health measures have been widely but unevenly used in conjunction with clinical data to support population health research.

## Introduction

### Nonclinical Determinants of Health

Nonclinical determinants of health, which refer collectively to the social, behavioral, and environmental factors and contexts that influence patient health outside of health care settings, are of growing importance to health care delivery and health policy. In terms of individual care, unmet needs related to nonclinical determinants of health can influence patient nonadherence to health care recommendations, limit patient-provider communication, exacerbate health conditions, and require significant time and organizational resources to address [1]. Moreover, nonclinical determinants of health needs are common. Estimates suggest that as many as half of primary care patients in the United States have unmet social needs [2,3]. For health care organizations, nonclinical determinants can inform risk stratification or patient segmentation efforts as health systems work to develop and target interventions and outreach appropriately [4,5]. From a health policy perspective, the nonclinical determinants of health illustrate disparities within the current US health system, many of which can only be addressed through policy interventions [6]. As such, health care organizations and policy makers are becoming more attentive to nonclinical determinants of health, as evidenced by initiatives from large, innovative health systems [7,8] and the specific screening and service linkage requirements in the Centers for Medicare and Medicaid Services Accountable Health Communities program [9].

### The Potential Utility of Combining Nonclinical Determinants with Clinical Data

Concurrent with growing interest in better addressing patients’ nonmedical issues is the exponential growth in availability of data sources that provide insight into the nonclinical determinants of health. Data potentially relevant to nonclinical determinants range from detailed individual-level observations (such as shopping behavior collected through a grocery store’s rewards application) to social networks and area-level measures of climate, built environment, or policy environment [10]. Numerous researchers and commentators see vast potential for indicators derived from these data to improve both the health care system and individual patient care [11-13]. In particular, the greatest gains might be realized using nonclinical determinants of health data in conjunction with clinical data sources, such as electronic health records (EHRs) and clinical registries [10]. These novel combinations of data could provide new insights into patient risk behaviors, factors complicating care delivery, population-level health assessment, health system evaluation, provider decision making, and more [14,15].

However, within this context of increasing availability of nonclinical determinants data, it is not widely understood which nonclinical determinants of health constructs and indicators are supported by the literature as useful for health services and policy research. Therefore, the purpose of this review was to characterize the state of the existing literature on the use of nonclinical health indicators in conjunction with clinical data. Specifically, we sought guidance on the domains of determinants (eg, socioeconomic status [SES] or built environment), data sources (eg, population registries and US Census data), and specific measures (eg, area median household income) that are necessary to characterize the nonclinical determinants of health for use in combination with clinical patient-level data. Review findings will be used to guide the development of a population health data commons that will link to comprehensive, community-wide clinical information.

## Methods

### Overview

We undertook a rapid review [16] of the published literature and relevant policy reports to support our institution’s broader project of developing the data architecture and governance policies necessary to create a data commons for clinical and nonclinical determinants of health information. Rapid reviews are literature reviews that are limited in scope and have a shorter time frame, typically up to 6 months [16]. Our institution’s broader initiative to develop a data architecture was in direct response to high-priority funding focused on the opioid epidemic and needed feedback from the review team within a few months. The information obtained from this rapid review informed the overall architecture of the system and corresponding metadata dictionaries and prioritized data for inclusion.

### Search Strategy

For the purpose of this review, we adopted a broad definition of nonclinical determinants of health that included individual-level behaviors, social contexts, physical environments, and health policies [6,17-19]. We operationalized *environment* to refer to different levels, such as a community, neighborhood, or family [20]. We took this approach to reflect the wide variation in potential use cases and research questions that could benefit from combined nonclinical determinants and clinical data.

### Study Eligibility

Articles and reports describing the effect of, the methods for, or the need for combining nonclinical determinants data with clinical data were eligible for inclusion. For this review, we defined clinical data as any patient-level data that were generated by health care encounters (eg, EHR data, claims, discharge records, immunization records, cancer or other disease registries, or genomic data). We did not consider public health surveys (eg, the National Health and Nutrition Examination Survey) to be clinical data. We did not limit study eligibility by study type and allowed for the inclusion of any study design and nonempirical expert commentaries. Only articles from peer-reviewed publications or reports from governmental agencies and grant-making organizations were eligible for inclusion. We limited the literature to English-language studies published in 2010 or after to reflect the widespread clinical information system adoption resulting from the introduction of the Health Information Technology for Economic and Clinical Health Act.

### Information Sources and Search Terms

The primary search concepts were nonclinical determinants of health and clinical data. Although there are numerous specific nonclinical determinants of health to make the search and screening manageable in the short amount of time available for a rapid review, we used keywords and Medical Subject Headings terms such as social determinants or factors, socioeconomic factors, behavioral factors, health disparities, environmental exposure, and exposome, a specialized term referring to the measure of all lifetime exposures of an individual and how these exposures relate to health. For the clinical data concept, we used terms such as EHR, electronic or computerized patient and medical data, medical order entry systems, and decision support systems. We selected these concepts based on several key reviews and reports [17,21,22]. Multimedia Appendix 1 provides the full search strategy. We searched 2 databases, MEDLINE (via Ovid) and Web of Science, in April 2018.

**Figure 1:**
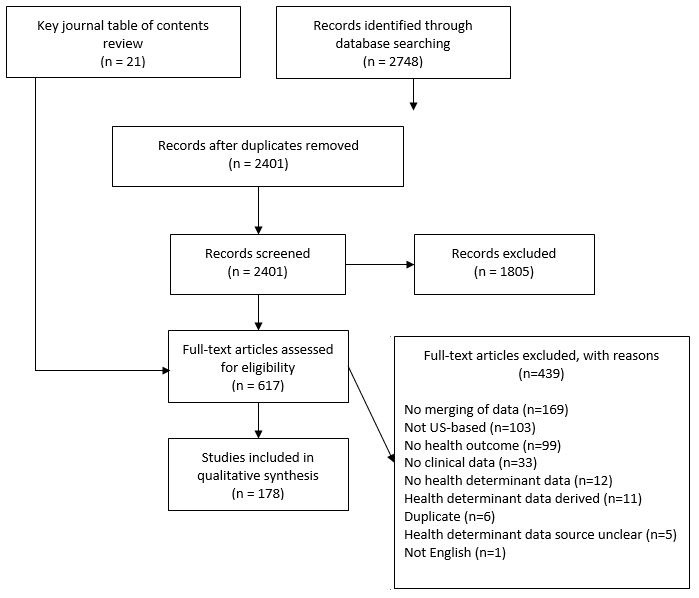
Diagram of articles reviewed for inclusion and qualitative synthesis.

All English-language articles from 2010 to April 2018 were exported to EndNote Version 8 citation management software (Clarivate Analytics). In addition, we manually reviewed the articles cited within selected articles, the table of contents from key journals (Multimedia Appendix 1), and the websites of the World Health Organization, Agency for Healthcare Research and Quality, National Institutes of Health, the National Academy of Medicine, and the Robert Wood Johnson Foundation for citations to relevant articles. We elected to search the websites of these governmental and nonprofit organizations in particular because of their focus on nonclinical determinants and population health. If the report summarized or presented previously published findings, we obtained those citations. The initial search yielded 2748 unduplicated records from the database search and 21 records from table of contents screening of key journals and website review (Figure 1).

### Study Selection

First, we screened the titles and abstracts of all records retrieved from the search. The primary goal of title and abstract screening was to exclude all non-US–based articles and articles with no indication of a focus on nonclinical determinants of health combined with clinical data. A total of 4 members of the research team first conducted a joint screening session on a random selection of citations to establish operational definitions and develop a cohesive screening approach. The team members then independently reviewed the title and abstract for each record to arrive at the included set. Our primary screening based on title and abstract resulted in 617 records for full-text review (Figure 1).

The research team members then independently read the full text of each article and determined its inclusion status on a randomly selected approximately 10% subsample from the 617 records identified from primary review. Agreement on inclusion status for the 10% subsample was kappa=0.70. The research team resolved differences by consensus in a joint reading session and independently reviewed the remainder of the articles. We retained articles for inclusion in the review if the article described the measurement or data source of at least one nonclinical determinant of health, which eliminated articles on technical architectures or database design issues that did not describe actual measurement. Owing to the study focus on nonclinical determinants and clinical data source linkages, we excluded articles in which the only nonclinical determinants of health measures were derived from clinical data (eg, insurance status or smoking history recorded within an EHR). Nonclinical determinants of health measures had to be derived from information systems, repositories, or collection methods apart from a clinical information system, including data from population surveys, epidemiologic registries, and US Census data. Furthermore, because our focus was on the use of these data for research, we limited inclusion to articles that used nonclinical determinants to describe, explore, or relate to a health outcome (eg, disease, condition, health status, and utilization). In addition, we reassessed each full-text article according to the exclusion criteria used for the initial title and abstract screening (ie, non-US–based articles or no focus on nonclinical determinants of health and clinical data). A total of 178 articles met the inclusion criteria after full-text review.

### Data Abstraction

An initial codebook was established, and after joint coding and discussion on a subset of articles to ensure consistent and calibrated data collection, the reviewers independently abstracted and coded relevant data elements from the full text using a standardized data collection instrument. We developed and refined the data collection instrument in light of the articles read jointly in the previous steps. We abstracted the measures, data sources, and level of measurement (individual or aggregate) for each nonclinical determinant of health reported. To organize the diverse set of reported nonclinical determinants into meaningful groups, we created domains based on a combination of existing conceptual frameworks and definitions [22-24]. We did not rely on any single framework to ensure that we captured a breadth of nonclinical determinants of health concepts and not only those of greatest interest to US policy makers and researchers. The domains are summarized in Table 1.

**Table 1:** Nonclinical determinants of health measurements by domain.

Nonclinical determinants domain	Example measures
Socioeconomic status and material conditions	Income, poverty, access to food, employment, living conditions, race and ethnicity, gender, insurance status
Behaviors	Smoking and tobacco use, diet, illicit substance use, alcohol use, medication adherence, physical activity
Built environment	Transportation, sidewalks, walkability, buildings
Natural environment	Air quality, pollution, climate, greenspace
Public policies	Health policies, social policies, laws, regulations
Health services and conditions	Access to health care, utilization, health literacy, disease prevalence
Social circumstances	Family, social support, caregivers, marital status, civic participation, community stigma

Furthermore, we grouped the reported study populations according to the key defining characteristics for inclusion in the study: geographic location, population focus (eg, Medicare enrollees, females only, and members of a specific racial or ethnic group), health condition of interest, or organization (ie, the study was focused on individuals who were part of the same health system or insurance plan). We also abstracted the study outcome, which we grouped into the broad categories of utilization, disease or health condition status, mortality, behaviors, risk scores, multiple outcomes, and all others. Other data elements that we abstracted include study design, type of clinical data source, use of census measures, and geographic level of measurement (for aggregate measures).

## Results

### Primary Findings

A total of 178 articles reported combining nonclinical determinants of health with clinical data (Multimedia Appendix 2 [25-145]). The most common source of clinical data was EHRs (62.9%; 112/178), followed by claims or discharge data (20.2%; 36/178) and disease registries (19.1%; 34/178). Approximately one-third of the articles (34.3%; 61/178) focused on utilization outcomes, and more than one-fourth (27.0%; 48/178) treated disease or condition status as the outcome. Among studies in which disease or condition status was the outcome, health status indicators were commonly related to body mass index or obesity, asthma, and diabetes. A common health condition (eg, diabetes and cancer) defined the study population for the majority of articles (53.9%; 96/178). One-fifth of studies included children in the study sample.

Included articles contained a mix of determinants measured at the aggregate (50.0%; 89/178) and individual (29.2%; 52/178) levels, with many studies using measures at both the aggregate and individual levels (20.7%; 37/178). Among the articles that included any aggregated measures, the geographic level tended toward smaller areas, with 43.6% (55/126) using areas smaller than a ZIP code (eg, a census tract) and 2.3% (3/126) using ZIP code–level measures. Articles with aggregated measures relied heavily on US Census Bureau data (81.7%; 103/126). Individual-level measures typically relied on questionnaires or supplemental screening (eg, studies by Sheppard et al [146] and Hall et al [147]). The literature appeared to be growing over time, as the number of articles meeting our inclusion criteria generally increased annually from ten articles in 2010 to almost forty in 2018.

The articles collectively reported on 744 different nonclinical determinants of health measures (Multimedia Appendix 3). The majority of articles reported using multiple measures as independent variables (Multimedia Appendix 4; however, several articles used existing or created new indices or composite measures (Multimedia Appendix 5). Most indices intended to summarize the SES and material conditions domain using various measures of income, employment, housing conditions, or other material deprivation. Below, we describe specific findings for each domain of nonclinical determinants.

### Socioeconomic Status and Material Conditions

Although the literature reflected all 7 of our identified nonclinical determinants of health domains, measures from the SES and material conditions domain dominated the literature, with 89.9% (160/178) of all articles including measures from this area. More than half of articles (57.9%; 103/178) used determinants from only a single domain, and if only 1 domain was reported, it was again largely from the SES and material conditions area. When articles reported on more than 1 domain, the additional domain was also most frequently an SES and material conditions measure.

Income, education, employment, and race and ethnicity-based measures were the most common approaches to representing this domain in the literature. Moreover, measures were highly variable and nuanced. For example, articles reported income as annual household income (eg, a study by Toledo et al [148]), mean household income (eg, a study by Seligman et al [149]), median household income (eg, a study by Grimberg et al [150]), or by various poverty measures (eg, studies by Ye et al [151], Kanzaria et al [152], and Patzer [153]). Similarly, multiple articles used the Gini coefficient to describe income inequality (eg, a study by Wallace [154]). Likewise, articles expressed employment status variously as employed (eg, a study by Shuman et al [155]), unemployment (eg, a study by Tanenbaum et al [156]), seasonal status (eg, a study by Castaneda et al [157]), job class (eg, a study by Eapen et al [158]), hours worked (eg, a study by DeMaria et al [159]), or employment rates by different age groups (eg, studies by Grimberg [150] and Wu [170]).

### Behaviors

All identified studies combining behavioral data with clinical data sources involved individual-level measurement (Multimedia Appendix 2), and nearly all (90%) in combination with EHR data. Current or historical substance, alcohol, or tobacco use [147,155,161-168]; self-care behaviors [169-171]; and self-reported physical activity levels and nutrition were commonly reported measures in the behaviors domain [147,172,173].

### Built Environment

The built environment domain included articles with measures ranging from a detailed description of neighborhood aesthetics [169] to traffic volume [174] and land use [175]. Measures were predominately at the aggregate level, and compared with articles with other domains, a higher proportion of articles considering built environment factors listed disease or condition status as outcomes.

### Natural Environment

Articles with measures related to the natural environment domain measured air pollution and quality [176-179], climate and altitude [179,180], and various hazardous exposures [147,181,182]. This small set of articles linked these measures mostly to EHR and registry data sources.

### Public Policies

The search strategy only identified 2 articles that linked public policy to clinical data sources. Achkar et al [183] combined state-level policy dates with prescription drug monitoring system usage data in an interrupted time series. Blosnich et al [184] used multiple measures to determine sociopolitical climate for hate crime protection in relationship to mental health status for transgender US veterans.

### Health Services and Conditions

The health services and conditions domain exhibited substantial variation in measures. Aggregate measures of health services and conditions included both the extent of a particular condition within an area (eg, infectious disease incidence rates [185], percent of population reporting a disability [186], or obesity prevalence [151]) and measures of the supply of providers and facilities within an area (eg, studies by Xiao et al [162], Beck et al [187], Roth et al [188], and Newman et al [189]). Measures reported on an individual basis included travel time and distance to health care provider [190,191]. Unique to this domain, variables such as emergency department overcrowding [192] and hospital quality [193,194] were measured at a facility level.

### Social Circumstances

Social circumstances was the second most common domain (25%) in the article set, and the most commonly employed measure was an indicator of a patient’s marital status, living arrangements, or family composition (eg, studies by Wu et al [170], Dupre et al [195], and Newgard et al [196]). Some measures moved toward deeper categorization of these arrangements by specifically looking at intimate partner violence or family conflict dynamics (eg, studies by Valentine et al [197] and Schuler et al [198]). Additional social circumstances reflected community stigma [190], social cohesion [169], self-reported social support [199], and structural racism [200].

## Discussion

### Summary of Findings

In this review, we sought to describe the extent to which existing research has combined numerous nonclinical determinants of health measures with different clinical datasets to explore a variety of health outcomes and conditions. Using domains derived from several established frameworks, we identified a comprehensive, but unevenly distributed, representation of nonclinical determinants domains across included studies. Measures related to SES and material conditions were most prevalent, followed by the closely related domain of social circumstances, reflecting the existing widespread availability and use of standard demographic measures such as marital status, education, race, ethnicity in public health surveillance. Although used less frequently in included studies, nonclinical determinants of health measures related to the domains of the built environment, the natural environment, and public policies may indicate a small but growing research base connecting these higher-level determinants with clinical data.

### Comment on Findings

We do not contend that any domain of nonclinical determinants is the most important; although different determinants arguably have varying importance or relative value, we could not assess the *value* of every reported measure within the scope of this study. Using the existing literature as a guide, measures reflective of SES and material conditions may be an appropriate initial focus for any work seeking to combine nonclinical determinants of health data with clinical data sources. The frequency with which this domain appears in the literature suggests it is a reasonable starting point for future work in this area that may be applicable to numerous different outcomes. Within this domain, income or lack of income was the most common measure, which is appropriate, given that multiple studies support income as an important determinant of health [201]. In addition, employment and education were common measures in the literature, conceptually distinct from income [202,203], and are supported by expert panels as key nonclinical determinants of health measures [204]. Conversely, a future area of focus may be where the literature is not well-developed (eg, housing and housing stability metrics). In addition, measures derived from race and ethnicity also appeared frequently in the literature. However, patient race and ethnicity are commonly recorded as data elements in routine clinical practice, and many sources of clinical data may already contain these data. Future work might explore the use and role of measures related to race and ethnicity that are less commonly recorded in clinical contexts, such as racism or cultural assimilation, but are significant nonclinical determinants of patient health.

Of potential further value, the SES and material conditions domain also had the largest number of indices and composite measures. Indices may be particularly useful for classification studies [203]. However, indices and composite measures have limitations as well. Indices developed in other countries may not be applicable to US populations, indices that were developed to measure specific constructs may not be applicable to all research studies, and indices and composite measures by design obscure the relationships between individual component measures [202,203].

Nonetheless, the literature in this area may be largely colored by the availability of nonclinical determinants of health data. As noted, most articles used data collected by the US Census Bureau. On the one hand, Census data have the dual advantages of longitudinal data and small area measurement. On the other hand, limitations exist with regard to the accuracy of some Census measures [205], and many measures are probability-based samples with errors around point estimates [206]. Problematically, the extent to which researchers consider the imprecision of measures in their analyses is not always immediately obvious. In addition, although clearly valuable, Census data do not readily, or thoroughly, extend to all nonclinical determinants of health domains. With Census data, nonclinical determinants of health related to SES and material conditions are well described, as are those related to the built environment and social circumstances, albeit to a lesser extent. However, other nonclinical determinants of health are largely unmeasurable using Census data (eg, person-level SES data and housing stability) and require either unique data collection or consideration of sources typically not used by health services and policy researchers. As more data sources become publicly and freely available, the distribution of studies using Census and non-Census sources may become more balanced and, in turn, highlight the growing potential to combine additional nonclinical determinants of health domains with clinical data. Similarly, the use of publicly available nonclinical determinants of health data sources is a reflection on the lack of relevant social determinant and social risk factors data currently captured in clinical information systems. Reliance on Census measures may further decrease as more individual-level measures of social risk are included in clinical datasets through wider implementation of social determinants of health (SDoH) screening tools [207,208] and collection of select social measurers in EHRs [209].

Articles included in this review reported both combining individually measured nonclinical determinants of health with clinical data sources (eg, measures of social support obtained by survey merged with EHR data) as well as combining aggregate, area-level measures with individual-level data sources through common geographical location (eg, applying area-level measures such as median household income to individual patients in an EHR, matched by patient address). In our study, the latter approach was more common. The various individual-level nonclinical determinants of health measures, such as transportation needs or social support, are consistent with the medical concept of a social history and express individual needs or resources [210]. The appropriateness of aggregate, area-level measures applied to individuals in the case of public policy is straightforward, as policy within a geographical jurisdiction is (at least theoretically) universally applied. However, the intention and reasoning for the differing levels of measurement linked to clinical data requires clear articulation for the other nonclinical determinants of health domains because characteristics of the aggregate cannot be attributed to the individual. For example, individuals residing within a high-poverty geographic area may not be themselves living in poverty; in fact, it is possible they are living well above the poverty line. When combined with individual-level data, aggregate or area-level measures are reflective of individuals’ situations and contexts but may not accurately capture individual circumstances [211-213]. As opportunities increase to link both aggregate-level and more comprehensive individual-level nonclinical determinants indicators (eg, derived from social media data, financial records, and surveys), the literature would benefit from a stronger articulation of the theoretical and methodological choices for levels of linkage with clinical data that to best explains the relationships between social determinants and health outcomes. Moreover, this growing data availability increases opportunities to empirically explore the value of combining individual and area-level SDoH data in explaining outcomes relevant at different levels of intervention, such as provider decision making, population health management, and research.

Previous reviews have commented on the frequent lack of theoretical justification for measurement choices and strategies [214,215]. The level of theoretical justification among the articles included in this review was highly variable. At one end of the spectrum, numerous articles provided extensive justification for selected constructs and measurement strategies (eg, studies by Valentine et al [197] and Schuch et al [216]). These articles tended to be those specifically interested in understanding the role of nonclinical determinants in influencing health outcomes. Other articles used nonclinical determinants of health measures as known confounding factors to be controlled and as such provided less explication and interpretation. Although many nonclinical determinants measures are, indeed, important and widely used confounders in analyses of health-related outcomes, it may be fruitful for future research in this area to provide more thoughtfully considered rationale for the selection of the nonclinical determinants used and how they may relate to the health outcome of interest. For example, income is reflective of the money received over a period of time by an individual or family. However, using income as a catch-all control measure may fail to capture the nuances and significant theoretical implications. For example, individuals and families have differing sources of income, such as salary versus investment, overall income fails to account for expenditures, and how income varies from others in society (ie, inequalities) may have stronger relationships with the health outcome of interest [217-219].

This review has several limitations. Although we grounded our categorization of the multiple nonclinical determinants of health in existing frameworks, other authors have grouped determinants in markedly different ways, thereby inhibiting direct comparisons with previous work. Differences may be most pronounced in our domain of SES and material conditions, which tended to be more expansive than in other frameworks. In addition, our search strategy undoubtedly undercounted articles describing the linkage of public policy and clinical data sources. Linking policy data to clinical records is very common in health policy, health services, and health economics research. However, our search strategy did not locate such articles because these disciplines tend to focus on the role of policy change, instead of framing public policy as a social determinant. Similarly, health services and conditions may also be undercounted because these measures may not be presented as measures of nonclinical determinants of health. Regardless, as our objective was focused on identifying measures, our smaller set of articles was still representative of the whole within these 2 domains. Also, our strategy excluded articles that did not merge nonclinical determinants of health data with clinical data. This requirement eliminated both articles that leveraged single information systems (such as EHRs) that already included nonclinical determinants of health measures, as well as articles focused solely on the measurement and impact of the nonclinical determinants of health. Similarly, our approach was focused on data that were linked to clinical information systems; as such, we do not discuss social factors or risks that may be inferred from clinical data (eg, homeless or transportation barriers) or that could be extracted from narrative texts in clinical documents. In addition, we do not comment on the appropriateness of measure choice, level of measurement, or linkage strategy. Finally, our review cannot be generalized to settings outside the United States.

### Conclusions

In conclusion, this review represents a comprehensive synthesis of existing attempts to link nonclinical determinants of health indicators with clinical data. Characterizing the work done so far in this area is an important first step in guiding future attempts to harness nonclinical determinants of health data for population health management initiatives. A better understanding of the types of determinants, data sources, and measures used successfully to integrate nonclinical determinants of health indicators with clinical and administrative health services data can help to shed light on feasibility, best practices, and future need in this important, emerging arena of research.

## Appendix

Multimedia Appendix 1 Search strategy.

Multimedia Appendix 2 Characteristics of literature on nonclinical determinants of health used in combination with clinical patient-level data.

Multimedia Appendix 3 Social determinant of health measures reported in the literature.

Multimedia Appendix 4 Combinations of social determinant domains reported by article.

Multimedia Appendix 5 Composite and index measures by social determinant of health domain.

## Abbreviations

EHR: electronic health record
SDoH: social determinants of health
SES: socioeconomic status
