# Supplementary material for: Combining Nonclinical Determinants of Health and Clinical Data for Research and Evaluation: Rapid Review
Source: JMIR Public Health Surveill. 2019 Oct 7;5(4):e12846. doi: 10.2196/12846 (PMC6803891; doi:10.2196/12846)
Supplement: Multimedia Appendix 1 [file publichealth_v5i4e12846_app1.pdf]

Multimedia Appendix 1: Search strategy

Web of Science

Date searched: 4/10/2018
TS=((“social determinants of health” OR ((socioeconomic OR social) NEAR/1 (determinant* OR factor*)) OR (behavior* NEAR/1 factor*) OR ((health or healthcare) NEAR/1 disparit*) OR exposome OR “environmental exposure”) AND (((clinical OR patient*) NEAR/1 data) OR "electronic health record*" OR ((electronic OR computerized) NEAR/2 (medical OR health OR patient)) OR "medical order entry systems*" OR "decision support system*"))
Limits:

- English language
- Document types: Article OR Editorial Material OR Letter OR News Item OR Note OR Proceedings Paper OR Review
- Publication date: 2010 – 2018 (Current)

TOTAL: 539 records

Medline Ovid

Date searched: 4/10/2018
(exp Social Determinants of Health/ or exp Socioeconomic Factors/ or (social adj2 determinant$).mp. or (social adj2 factor$).mp. or (behavior$ adj2 factor$).mp. or Social conditions/ or Healthcare Disparities/ or Health Status Disparities/ or (health adj3 disparities).mp. or Exposome.mp. or Environmental Exposure/) AND (((clinical OR patient*) adj2 data) OR exp Electronic Health Records/ OR ((electronic or computerized) adj3 (medical or health or patient) adj3 record*) OR Medical Order Entry Systems/ OR Decision Support Systems, Clinical/)
Limits:

- English language
- Publication date: 2010 – current

Total: 2209 records

Journal Table of Contents Searched

- Health Affairs
- New England Journal of Medicine
- Journal of the American Medical Association
- Applied Clinical Informatics
