# Supplementary material for: Combining Nonclinical Determinants of Health and Clinical Data for Research and Evaluation: Rapid Review
Source: JMIR Public Health Surveill. 2019 Oct 7;5(4):e12846. doi: 10.2196/12846 (PMC6803891; doi:10.2196/12846)
Supplement: Multimedia Appendix 2 [file publichealth_v5i4e12846_app2.pdf]

Multimedia Appendix 2. Characteristics of literature on nonclinical determinants of health used in combination with clinical patient-level data.

Characteristics		Total (N=178)	Social determinant domains						
			Socioeconomic status and material conditions (n=161)	Behaviors (n=20)	Built environment (n=25)	Natural environment (n=8)	Public policies (n=2)	Health services and conditions (n=20)	Social circumstances (n=45)
**Clinical data source n (%)**									
	EHR[a]	112 (62.9)	100 (62.1)	18 (90)	16 (64)	6 (75)	1 (50)	17 (85)	35 (78)
	Registry	34 (19.1)	32 (19.9)	2 (10)	4 (16)	2 (25)	0 (0)	2 (10)	7 (16)
	Claims or discharge	36 (20.2)	18 (11.2)	0(0)	2 (8)	0 (0)	0 (0)	1 (5)	0 (0)
	Various	21 (11.8)	11 (6.8)	0 (0)	3 (12)	0 (0)	1 (50)	0 (0)	3 (7)
**Outcomes (%)**									
	Utilization	61 (34.3)	60 (37.3)	2 (10)	5 (20)	2 (25)	1 (50)	7 (35)	11 (24)
	Disease or health condition status	48 (27.3)	43 (26.7)	6 (30)	10 (40)	4 (50)	0 (0)	3 (15)	11 (24)
	Mortality	14 (7.9)	21 (13.0)	2 (10.0)	2 (8)	1 (13)	0 (0)	2 (10)	3 (7)
	Risk scores	7 (3.9)	16 (9.9)	1 (5)	1 (4)	0 (0)	0 (0)	2 (10)	2 (4)
	Behaviors	5 (2.8)	11 (6.8)	4 (20)	0 (0)	0 (0)	0 (0)	0 (0)	4 (9)
	Multiple	27 (15.2)	6 (3.7)	4 (20)	1 (4)	0 (0)	1 (50)	4 (20)	7 (16)
	Other	16 (8.9)	3 (1.9)	1 (5)	1 (4)	1 (13)	0 (0)	2 (10)	7 (16)
**Unit of focus (%)**									
	Condition	96 (53.9)	92 (57.1)	16 (64)	10 (40)	2 (25)	0 (0)	15 (75)	30 (67)
	Demographic	32 (17.9)	24 (14.9)	3 (12)	6 (24)	3 (38)	1 (50)	0 (0)	6 (13)
	Organizational	27 (15.2)	24 (14.9)	5 (25)	3 (12)	2 (25)	0 (0)	3 (15)	4 (9)
	Geographic	23 (12.9)	21 (13.0)	0 (0)	6 (24)	1 (13)	1 (50)	3 (15)	5 (11)
Children included (%)		37 (20.8)	34 (21.1)	0 (0)	9 (36)	4 (50)	0 (0)	3 (15)	6 (13)
**Level of measurement (%)**									
	Aggregate	89 (50.0)	81 (50.3)	0 (0)	20 (80)	5 (63)	2 (100)	9 (45)	7(16)
	Individual	52 (29.2)	48 (29.8)	20 (100)	5 (20)	3 (38)	0 (0)	10 (50)	38 (84)
	Both	37 (20.8)	32 (19.9)	0 (0)	0 (0)	0 (0)	0 (0)	1 (5)	0 (0)

[a]EHR: electronic health record.
