# Supplementary material for: Combining Nonclinical Determinants of Health and Clinical Data for Research and Evaluation: Rapid Review
Source: JMIR Public Health Surveill. 2019 Oct 7;5(4):e12846. doi: 10.2196/12846 (PMC6803891; doi:10.2196/12846)
Supplement: Multimedia Appendix 3 [file publichealth_v5i4e12846_app3.pdf]

Multimedia Appendix 3: Social determinant of health measures reported in the literature.

Measure	References
Acculturation	[38]
Adverse childhood event	[76]
Age	[34, 40, 43, 48, 49, 51, 60, 67, 71-73, 75, 76, 78, 93-120]
Air quality	[30, 57, 58]
Alcohol use	[26, 34, 44, 45, 47, 74]
Arsenic exposure	[61]
Barriers to treatment	[69]
Built environment index	[93]
Climate	[58, 59]
Community resources	[121, 122]
Community stigma	[69]
Composite SES Index	[39, 54, 78, 94, 116, 123-136]
Cost of living	[137]
Crime rate	[66]
Depression	[74]
Diabetes rate	[30]
Dietary behavior (caffeine)	[26]
Dietary behavior (food intake)	[51]
Dietary behavior (food selection)	[138]
Dietary behavior (servings)	[52]
Dietary behavior (sugary beverages)	[52]
Disability	[139]
Disability rate	[65]
Distance to facility	[32, 69, 71, 73, 140]
ED overcrowding	[71]
Education (college degree)	[37, 74, 140-143]
Education (high school degree)	[29, 37, 142, 144, 145]
Education (less than high school)	[132, 146]<br>[147, 148]
Education (parents)	[137, 149]
Educational attainment	[25, 30, 32, 34, 35, 38, 43-47, 49, 51, 57, 65, 67-69, 73, 75, 76, 78, 96, 98-100, 102, 107, 108, 110-114, 119, 121, 126, 130, 138, 150-161]
Emergency food assistance	[113, 121, 162, 163]

Employment	[29, 32, 34, 44, 50, 51, 68, 69, 73-75, 99-101, 111-113, 130, 138, 145, 155, 164, 165]
Employment (hours worked)	[38]
Employment (type)	[153]
Employment (unemployment)	[30, 35, 57, 67, 121, 132, 148, 152, 163, 166-168]
Employment (white collar)	[37]
Environmental exposures	[60]
Family cohesiveness	[77]
Family conflict resolving	[77]
Family expressiveness	[77]
Financial burden	[73]
Financial status	[49]
Fitness & Rec Centers	[67]
Food environment	[30, 121, 169]
Food environment (good food access)	[170]
Food environment (proximity)	[171]
Food environment (restaurants)	[172]
Food environment (retail foods)	[143]
Food environment (supermarkets)	[172]
Food environment (farmer's markets)	[67]
Food insecurity	[30, 48]
Foreclosure rates	[168]
Foregone medical care	[113]
Gender	[29, 32, 34, 40, 48, 49, 60, 67, 72, 75, 78, 93-95, 97, 98, 101, 103-108, 110, 112, 115, 117-120, 122, 126, 138, 150, 164, 173]
Gini coefficient	[30, 33, 119, 174]
Greenspace	[121]
Health insurance	[30, 32, 34, 41, 45, 48, 59, 68-71, 74, 96, 98, 99, 101-106, 112, 119, 121, 126, 127, 137, 152, 155, 173, 175, 176]
Health insurance (Medicaid dual eligible)	[177]
Health insurance (Medicaid)	[132, 178-180]
Health insurance (uninsured)	[132]
Health literacy	[35, 74, 78, 111, 112, 164]

Health self-efficacy	[74]
Health status	[121]
Healthcare access	[68, 155]
Home ownership	[26, 137, 146, 148, 168, 181]
Homelessness	[127]
Hospital location	[106]
Hospital quality	[72]
Household allergens	[182]
Household composition	[57, 75, 113, 119, 138, 146, 163, 166]
Household density	[54]
Housing conditions	[26, 37, 57, 66, 93, 120-122, 138, 143, 153, 168, 181, 183, 184]
Incarceration rates	[122]
Income	[51, 59, 60, 72, 73, 78, 96, 98, 101, 106, 111, 113-115, 149, 156, 162, 164, 185-188]
Income (household)	[27, 35, 38, 48, 69, 75, 108, 110, 112, 120, 153, 189]
Income (mean household)	[28, 158, 190]
Income (median household)	[29, 37, 41, 46, 57, 65, 67, 70, 97, 107, 109, 118, 119, 121, 130, 132, 137, 141-148, 150, 152, 154, 157, 159-161, 166, 169, 171, 175, 177, 191-205]
Income (per capita)	[62, 65, 190]
Income inequality	[79]
Influenza-like illness prevalence	[58]
Intersection density	[54]
Lack of personal resources	[69]
Land mass	[75]
Land-use mix	[54]
Language	[71, 93, 97, 112, 121]
Language (does not speak English)	[206]
Language (English proficiency)	[30, 111, 112]
Language (preferred)	[173]
Language (primary)	[27, 75]
Lead paint exposure	[120]
LGBT Municipality Equality Index	[63]
Life stressors	[74]
Lives in large village of >500 persons	[184]
Lives independently	[50]

Living arrangements	[49, 74]
Marital status	[27, 34, 41, 44, 47, 48, 51, 69, 73, 74, 95, 97-102, 106-109, 113, 114, 116, 117, 149, 152, 153, 164, 173]<br>[38, 165, 207]
Means of transportation	[69]
Medically underserved areas	[66]
Medication adherence	[44, 74]
Medication usage	[66]
Migrant vs. seasonal worker status	[36]
Mobility	[93]
Nativity	[65]
Nativity (citizenship)	[30]
Nativity (foreign born)	[51, 57]
Neighborhood attractiveness	[48, 121]
Noise	[48]
Number of health care providers	[41]
OB vs. midwife	[27]
Obesity rate	[30]
Parcel location	[138]
Parity	[114]
Parks	[30]
Particulate matter exposure	[55, 56]
Partner violence	[76]
Perceived discrimination	[25, 44]
Perceived safety	[121]
Physical activity	[26, 51, 52, 54, 169]
Population density/size	[54, 67, 143, 168, 170, 194, 197]
Positive cultures	[64]
Poverty	[30-32, 35, 57, 65-68, 103, 104, 119, 132, 140, 142, 145-148, 152, 162, 163, 168, 170, 174, 181, 184, 195, 208, 209]
Precipitation	[59]
Prescription policy	[62]
Presenteeism	[210]
Quality of life	[44, 50, 210]
Race/ethnicity	[30, 32, 34, 40, 48, 51, 59, 60, 67, 70-72, 75, 76, 93-101, 103-115, 124, 149, 150, 155, 162, 164, 169, 192]<br>[29, 43, 57, 73, 116-120, 130, 211]

Race/ethnicity (ancestry)	[126]
Race/ethnicity (composition)	[189, 212]
Race/ethnicity (Hispanic)	[173]
Race/ethnicity (maternal)	[79]
Race/ethnicity (percent AA or Hispanic)	[174]
Race/ethnicity (percent African American)	[119, 140, 143, 188]
Race/ethnicity (percent Hispanic)	[119, 140]
Race/ethnicity (percent nonwhite)	[132]
Race/ethnicity (percent White)	[168]
Racism	[79]
Relationship status	[43, 46]
Rurality	[30, 32, 102, 118, 132, 135, 141, 178, 207, 213-215]
Rurality (farm house presence)	[41]
Rurality (location)	[216]
Rurality (resides on a farm)	[26]
Rurality (urbanicity)	[131, 144]
Segregation	[212]
Self-management behaviors	[48-50]
Service utilization	[138]
Sexual history	[38]
Sexual minority	[76]
Sexual orientation	[43]
Social cohesion	[48]
Social disorganization index	[54]
Social support	[44, 47, 48, 74, 78]
Substance abuse	[40, 44, 46, 76]
Suicidality	[76]
Sun exposure	[26]
Testing rates	[64]
Tobacco exposure	[26]
Tobacco use	[26, 34, 41, 43-45, 47, 74, 110, 121]
Traffic volume	[217]
Transience	[127]
Transportation	[52, 121]
Travel time to clinic	[69]
Trust	[25]
Walkability	[48]

Work productivity	[139]
