# Supplementary figures and images for: Combining Nonclinical Determinants of Health and Clinical Data for Research and Evaluation: Rapid Review
Source: JMIR Public Health Surveill. 2019 Oct 7;5(4):e12846. doi: 10.2196/12846 (PMC6803891; doi:10.2196/12846)

#### Multimedia Appendix 4. Combinations of social determinant domains reported by article.

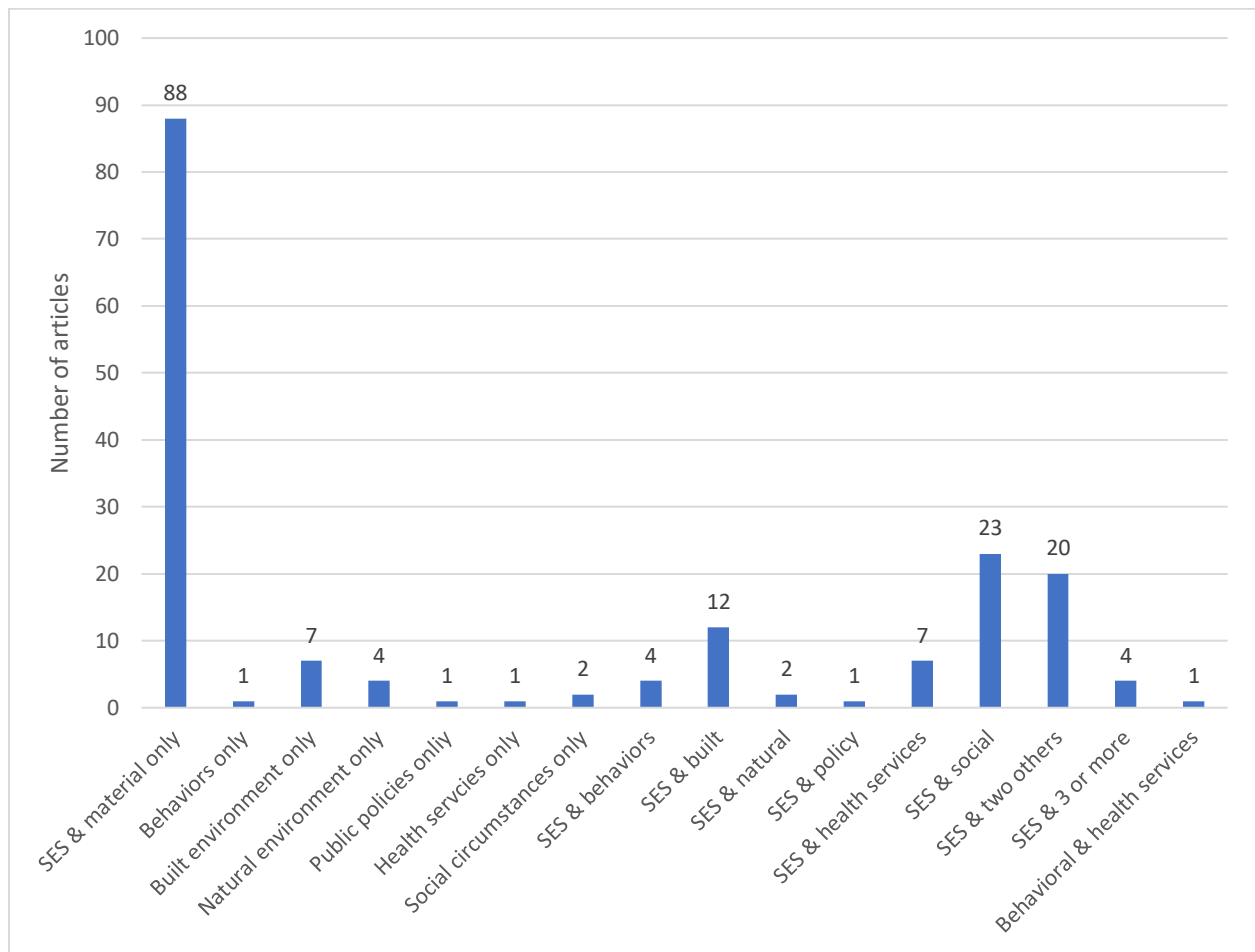

Supplement: Multimedia Appendix 4 [file publichealth_v5i4e12846_app4.pdf]
