# Supplementary material for: Combining Nonclinical Determinants of Health and Clinical Data for Research and Evaluation: Rapid Review
Source: JMIR Public Health Surveill. 2019 Oct 7;5(4):e12846. doi: 10.2196/12846 (PMC6803891; doi:10.2196/12846)
Supplement: Multimedia Appendix 5 [file publichealth_v5i4e12846_app5.pdf]

Multimedia Appendix 5. Composite and index measures by social determinant of health domain

Reference	Domain	Components
Finks et al. 2011[136]	socioeconomic status & material conditions	• log of the median household income<br>• log of the median value of housing units<br>• percent households receiving interest, dividend, or net rental income<br>• percent adults 25 years of age or older who had completed high school<br>• percent adults 25 years of age or older who had completed college<br>• percent employed persons 16 years of age or older in executive, managerial, or professional specialty occupations
Tumin et al. 2017[123]	socioeconomic status & material conditions	Socioeconomic advantage indicators<br>• median household income<br>• median value of owner-occupied housing units<br>• percent households with interest, dividend, or net rental income -percent workers employed in management, business, science, and arts occupations<br>• percent residents >25 years of age with a high school diploma<br>Index of concentration at the extremes<br>• concentration of wealthy families (income > $75,000)<br>• concentration of poor families (below federal poverty line)<br>Index of dissimilarity<br>• percent white or African American households required to achieve integration
Takahashi et al. 2016 [125]	socioeconomic status & material conditions	• housing value<br>• housing square footage<br>• number of bedrooms<br>• number of bathrooms
Schwartz et al. 2011[54]	socioeconomic status & material conditions / social circumstances	Modified Townsend Index<br>• percent with less than high school education<br>• percent not in labor force<br>• percent in poverty<br>• percent on public assistance<br>• percent unemployed<br>• percent that does not own a car<br>Social disorganization index<br>• percent of households headed by women<br>• percent divorced<br>• percent owner-occupied housing<br>• percent residential instability
Sanchez et al. 2012[126]	socioeconomic status & material conditions	Graffar scale of socioeconomic level<br>• family monthly income<br>• occupation of the head of the household<br>• percentage of family income spent on food<br>• type and characteristics of residence (owner occupied, rented, or shared with extended family)<br>• place of residence<br>• presence of chronic illnesses in other family members
Parker et al. 2012[78]	socioeconomic status & material conditions	Deprivation index<br>• percent males in management and professional occupations<br>• percent crowded housing<br>• percent households in poverty<br>• percent female headed households with dependents<br>• percent of households on public assistance<br>• households earning <$30,000 per year<br>• percent earning less than a high school education<br>• percent unemployed
Nijhawan et al. 2012[127]	socioeconomic status & material conditions	• median household income<br>• percent households below federal poverty level<br>• percent adults (>18) with less than college or higher education<br>• percent non-white population
Nau et al. 2015[128]	socioeconomic status & material conditions	• percent with less than high school education<br>• percent unemployed<br>• percent not in labor force<br>• percent receiving public assistance<br>• percent households without a car
Keegan et al. 2015[116]	socioeconomic status &	CDC index<br>• education index

	material conditions	• median household income<br>• percent living below 200% federal poverty level<br>• percent blue-collar workers<br>• percent older than 16 in workforce and unemployed<br>• percent without a car<br>• median rent<br>• median house value
Martinez et al. 2016[129]	socioeconomic status & material conditions	• education<br>• occupation<br>• employment<br>• household income<br>• poverty<br>• rent<br>• house values
Flood et al. 2015[131]	socioeconomic status & material conditions	Economic hardship index<br>• percent housing units with more than one person/room<br>• percent households below federal poverty level<br>• percent older than 16 unemployed<br>• percent older than 25 without high school education<br>• dependency (percent greater than 64 and less than 18 years old)<br>• per capita income
Tomayko et al. 2016[124]	socioeconomic status & material conditions	Economic hardship index<br>• percent housing units with more than one person/room<br>• percent households below federal poverty level<br>• percent older than 16 unemployed<br>• percent older than 25 without high school education<br>• dependency (percent greater than 64 and less than 18 years old)<br>• per capita income
Tomayko et al. 2015[94]	socioeconomic status & material conditions	Economic hardship index<br>• percent housing units with more than one person/room<br>• percent households below federal poverty level<br>• percent older than 16 unemployed<br>• percent older than 25 without high school education<br>• dependency (percent greater than 64 and less than 18 years old)<br>• per capita income
Finney Rutten et al. 2017[132]	socioeconomic status & material conditions	• low median income<br>• high percent unemployment<br>• high percent non-college education<br>• high Medicaid insurance<br>• high poverty
Dalton et al. 2017[133]	socioeconomic status & material conditions	• percent white non-Hispanic<br>• percent high school degree<br>• percent Medicaid<br>• percent ages 18-64<br>• median income<br>• percent households below federal poverty level<br>• percent children in households receiving supplemental security income<br>• percent cash public assistance income, food stamps, or Supplemental Nutrition Assistance program<br>• percent households headed by unmarried mother
Collins et al. 2013[134]	socioeconomic status & material conditions	• percent nonwhite<br>• percent foreign born<br>• percent not completing high school<br>• percent below poverty<br>• median income
Casey et al. 2013[135]	socioeconomic status & material conditions	• percent less than high school education<br>• percent not in labor force<br>• percent in poverty<br>• percent on public assistance<br>• percent civilian unemployment<br>• percent does not own a car
Schuch et al. 2017[93]	built environment	Environmental lead index<br>• peeling paint<br>• original features<br>• exposed soil<br>• water damage

		• gravel driveway<br>• dripline exposed<br>• play space<br>• gardening activity
Gebauer et al. 2017[39]	socioeconomic status & material conditions	• percent households with income below poverty level<br>• percent households receiving public assistance<br>• percent households with an annual income below $35,000<br>• percent adult males ages 20-64 not in the labor force<br>• percent adult males ages 20 and older with less than a high school education<br>• log median household income<br>• log median value of single family homes
Blosnich et al. 2016[63]	public policies	Municipality Equality Index<br>• nondiscrimination law that includes sexual orientation and gender identity<br>• recognition of same sex relationships<br>• equal benefits and protections for LGBT individuals employed by the municipality<br>• inclusion of LGBT constituents in city services and programs<br>• inclusion of LGBT issues in law enforcement and participation in the Federal Bureau of Investigation hate crimes statistics<br>• city leadership's relationship with the LGBT community
